# Comparison of the Four Anthropometric Indexes and Their Association With Stroke: A Population-Based Cross-Sectional Study in Jilin Province, China

**DOI:** 10.3389/fneur.2019.01304

**Published:** 2019-12-10

**Authors:** Peng Zhang, Xin Sun, Hang Jin, Fu-Liang Zhang, Zhen-Ni Guo, Yi Yang

**Affiliations:** ^1^Department of Neurology, The First Hospital of Jilin University, Clinical Trial and Research Center for Stroke, Changchun, China; ^2^Department of Neurology, The First Hospital of Jilin University, Changchun, China

**Keywords:** stroke, ischemic stroke, waist-height ratio, body fat distribution, body mass index, waist circumference

## Abstract

**Background:** Stroke is a leading cause of long-term adult disability and death in China, and primary prevention is essential for stroke. The aim of this study is to compare the four anthropometric indexes (body mass index, waist circumference, waist-to-hip ratio, and waist-to-height ratio) and identify the index that is most closely related to stroke in areas with high incidence of stroke.

**Methods:** A total of 4,052 participants aged 40 years or older were selected by the multistage stratified cluster sampling method in Dehui City in Jilin province, China. Face-to-face interviews and physical examinations were conducted to collect the participants' information. Descriptive data analyses were conducted. Multivariable logistic analyses were used to explore the adjusted association between stroke and body fat measuring indexes.

**Results:** For body mass index (adjusted odds ratio (OR): 1.13, 95% confident interval (CI): 0.77–1.65), waist circumference (adjusted OR: 1.32, 95% CI: 0.88–1.99), and waist-to-hip ratio (adjusted OR: 1.34, 95% CI: 0.92–1.93), the patients in the highest quartile did not have a higher risk of stroke than those in the lowest quartile. For waist-to-height ratio, the patients in the highest quartile were more likely have a stroke than those in the lowest quartile (adjusted OR: 1.81, 95% CI: 1.16–2.82). The area under the curve of waist-to-height ratio, waist-to-hip ratio, waist circumference, and body mass index for stroke were 0.627 (95% CI: 0.595–0.659; sensitivity: 65.10%; specificity: 56.00%), 0.596 (95% CI: 0.593–0.629; sensitivity: 69.90%, specificity: 45.90%), 0.612 (95% CI: 0.579–0.644; sensitivity: 52.70%, specificity: 64.70%), and 0.548 (95% CI: 0.514–0.583; sensitivity: 57.90%, specificity: 51.10%), respectively.

**Conclusions:** The waist-to-height ratio was more closely related to the prevalence of stroke than body mass index, waist circumference, and weight-to-hip ratio among the study participants aged ≥ 40 years.

## Introduction

Stroke is one of the main cause of disability and death in Chinese adults (1, 2). From 2002 to 2013, the incidence of stroke continued to rise (3) and will likely present an even greater burden of disease over the next few decades in the absence of effective primary prevention measures in China (4). The risk factors for stroke substantially contribute to its incidence such as diabetes, hypertension, obesity, smoking, and low physical activity. Obesity is closely associated with the risk of hypertension, abnormal body fat accumulation may affect the normal function of blood vessels (5). Therefore, early control of abnormal fat accumulation is critical for reducing stroke risk. However, the appropriate way to describe the state of abnormal body fat accumulation associated with stroke risk is unclear.

In general, the state of body fat accumulation can be described by four indexes: waist circumference (WC), body mass index (BMI), waist-to-height ratio (WHtR), and waist-to-hip ratio (WHR). BMI is calculated as weight/height (2). WHtR is the ratio of WC to height. WHR is the ratio of WC to hipline. A multinational study suggest that these indexes may have different values in predicting the risk of stroke, with a large proportion of them being Chinese (6). However, Jilin province is located in the northeast of China, and has a higher incidence of stroke (365/100,000 person-years) than other areas of China (326/100,000 person-years in Central China; 316/100,000 person-years in Northwest China; 275/100,000 person-years in North China; 232/100,000 person-years in East China; 154/100,000 person-years in South China; 153/100,000 person-years in Southwest China) (7). The epidemiological characteristics of Jilin Province may be different from other areas in China. Study comparing the value of the four anthropometric indexes and its association with the prevalence of stroke in this area are rare. It is still unclear whether the relationship between stroke and obesity indicators is different between areas with high incidence of stroke and areas with low incidence of stroke. Comparing the relationship between stroke and the four anthropometric indexes is important for guiding the primary prevention of stroke in Jilin province. This study is aimed to compare the four anthropometric indexes and identify the index that is most closely related to stroke in areas with high incidence of stroke.

## Materials and Methods

### Study Design and Population

In 2016, we conducted a population-based cross-sectional study among residents in Dehui, Jilin province, China. Residents aged 40 years or older and had lived in Dehui for more than 6 months were eligible for screen. Multistage stratified cluster sampling method was used for sample selection. Firstly, 30 villages (rural) and 10 towns (urban) in Dehui were randomly selected using probability proportional to size (PPS) sampling. Secondly, 4 or 5 communities were sampled from each villages and towns using PPS. Finally, one adult resident aged 40 years or older were randomly selected from each household in the selected community. The required sample size (N) was calculated based on a 2.37% prevalence (p) of stroke among adults aged 40 years or older in China (8), using the formula *N* = (${\text{Z}}_{\text{α}}^{2}$pq)/d^2^ (where *Z*_α_ = 1.96, α = 0.05, *q* = 1-p, and *d* = 0.2p). Considering a 10% loss rate, we recruited 4,445 subjects, of which 4,100 completed the survey. The response rate of this survey was 92.23%. Finally, 4,052 subjects with complete information were included in this study.

### Data Collection

Data were collected by face-to-face interview or body examination. The data included three parts: general information (such as socio-demographic characteristics), anthropometric measurement information (such as waist circumference, height, and weight), and blood biochemical information (such as blood glucose and blood lipid levels). The researchers received uniform training before data collection. The information of the participants was collected in accordance with the same questionnaire guidance.

### Ethics Approval

This study was approved by the human ethics and research ethics committee of the First Hospital of Jilin University (Approval No: 2015-R-250), and written informed consent was obtained from all of the participants.

### Measurements

The subjects were asked to wear light clothing and no shoes when measuring their height and weight by an electronic scale (OMRON HNH-219). The measurement accuracy of height and weight is 0.1 cm and 0.1 kg, respectively. Waist circumference was measured by placing the tape around 0.5–1.0 cm above the navel level with the subjects breathing naturally and wearing thin clothing. Hipline was measured at the point yielding the maximum circumference over the buttocks. An electronic sphygmomanometer (OMRON HEM-7200) was used to measure the systolic and diastolic pressure of the subjects after 20 min of rest. The blood samples were collected after the subjects fasted for at least eight hours and were transported to a clinical laboratory (Changchun Kingmed Center for Clinical Laboratory Co. Ltd.) by cold chain to measure blood biochemical indicators (such as blood glucose and blood lipid levels). All of the blood samples were processed within 8 h after collection.

### Definitions

Stroke was defined according to the World Health Organization (WHO) criteria: rapidly developing clinical symptoms of brain dysfunction that last for 24 h or longer or leading to death, with no apparent cause except for vascular origin. If the subjects had a history of stroke, they were asked to provide detailed medical records containing patient history, neurological imaging, and so on. Hemorrhagic or ischemic stroke was determined by medical records or neuroimaging provided by patients with a history of stroke. Hyperlipidemia was defined according to the following points: TC ≥ 5.18 mmol/L or TG ≥ 1.70 mmol/L or HDL-C < 1.04 mmol/L or LDL-C ≥ 3.37 mmol/L or a previous diagnosis of hyperlipidemia by a physician (9). Definition of diabetes was based on abnormal fasting plasma glucose (FPG ≥ 7.0 mmol/L) or physician's diagnosis (10). Hypertension was defined according to the following points: systolic ≥ 140 mmHg or diastolic ≥ 90 mmHg and/or self-reported hypertension (11). Smoking status was divided into three groups: current smoking, former smoking, and never smoking. Current smoking was defined as consuming any kind of tobacco products at the time of the interview. Those who had quit smoking for more than 3 months were defined as former smokers. Never smokers are those who had never smoked or who had smoked fewer than 100 cigarettes in their entire life (12). Drinking was defined as consuming more than 42 g pure alcohol per day or 98 g pure alcohol per week (13). Physical activity level was determined by questionnaire in this study. Participants were asked how many times do they exercised each week (including industrial and agricultural labor). How long does it last each time? Physical exercise (including industrial and agricultural labor) less than 3 times a week, each time lasting less than 30 min, was defined as irregular exercise. All of the included participants underwent electrocardiogram (ECG) examination, and coronary heart disease was judged by physicians based on the results.

### Statistical Analysis

Data were described according to the distribution characteristics of the data. The difference of stroke prevalence among different groups were compared by Chi-squared test. The value of BMI, waist circumference, waist-to-hip ratio, and waist-to-height ratio were divided into quartiles (Q1: < 25%; Q2: 25%~; Q3: 50%~; and Q4: 75%~). The median of each quartile was used for a linear trend test. The receiver operating characteristic (ROC) curve was used to compare the area under the curve (AUC) between the four anthropometric indexes. Comparison of two ROC curves was based on the method of Delong et al. (14). Multivariable logistic analyses were used to identify the association between stroke and the body fat measuring indexes. Three models with different covariates adjusted were used for sensitivity analysis. Covariates were not adjusted in model 1, and possible covariates were adjusted as much as possible in model 3. We aimed to get the upper and lower limit range of OR value through sensitivity analysis. Age, sex, area, education, smoking, drinking, hypertension, diabetes, hyperlipidemia, coronary heart disease, family history of stroke, and regular exercise were considered as covariates in model 3, as the final result. Seven of the ten potentially modifiable risk factors identified by the INTERSTROKE study were included in our study (15). Although apolipoprotein (Apo)B/ApoA1 ratio was not considered in our study, hyperlipidemia was included as a covariate in our study. Dietary and psychological factors were not considered in our study, because too many questions about dietary and psychological scale in the survey will reduce the response rate of participants and increase the difficulty of research. The ORs of each anthropometric variable for a one standard deviation increase were also calculated by multivariable logistic regression model. All of the tests were two-tailed, and *P* < 0.05 was considered statistically significant. All calculations were made using SPSS 22.0 (IBM Corp., Armonk, NY, USA) or MedCalc (MedCalc, Mariakerke, Belgium).

## Results

A total of 4,052 participants over 39 years of age were included. The mean age of the included participants was 54.85 ± 9.30 years. The prevalence of stroke was 7.2%, and all patients were historical cases. No patients were newly diagnosed with stroke during the survey. Among them, 91.7% were ischemic stroke, 8.3% were hemorrhagic stroke. The prevalence of stroke was varied among the different sexes, ages, areas, and other characteristics of the participants (Table 1). The prevalence of stroke decreased with education and increased with age.

**Table 1:** Characteristics of the included participants aged 40 years or older (*n* = 4,052).

**Characteristics**	**Stroke**		**χ^2^**	**p**
	**Yes**	**No**		
**Sex**			23.795	<0.001
Male	156 (9.6)	1,463 (90.4)		
Female	136 (5.6)	2,297 (94.4)		
**Age (years)**			163.425	<0.001
40~	26 (1.9)	1,350 (98.1)		
50~	81 (5.9)	1,291 (94.1)		
60~	133 (13.2)	876 (86.8)		
70~	52 (17.6)	243 (82.4)		
**Area**			4.761	0.029
Urban	131 (6.3)	1,936 (93.7)		
Rural	161 (8.1)	1,824 (91.9)		
**Education**			31.148	<0.001
Primary school and below	146 (10.1)	1,300 (89.9)		
Junior middle school	106 (6.2)	1,590 (93.8)		
Senior middle school	24 (4.5)	513 (95.5)		
College and above	16 (4.3)	357 (95.7)		
**Smoking**			24.568	<0.001
Current	137 (10.0)	1,236 (90.0)		
Never	122 (6.0)	1,903 (94.0)		
Former	33 (5.0)	621 (95.0)		
**Drinking**			3.010	<0.001
Yes	90 (8.4)	984 (91.6)		
No	202 (6.8)	2,776 (93.2)		
**Hypertension**			82.167	<0.001
Yes	242 (10.4)	2,093 (89.6)		
No	50 (2.9)	1,667 (97.1)		
**Diabetes**			11.096	0.001
Yes	45 (11.3)	353 (88.7)		
No	247 (6.8)	3,407 (93.2)		
**Hyperlipidemia**			18.173	<0.001
Yes	219 (8.5)	2,351 (91.5)		
No	73 (4.9)	1,409 (95.1)		
**Coronary heart disease**			17.841	<0.001
Yes	37 (13.6)	3,525 (86.4)		
No	255 (6.7)	235 (93.3)		
**Family history of stroke**			16.034	<0.001
Yes	132 (9.4)	1,265 (90.6)		
No	160 (6.0)	2,495 (94.0)		
**Regular exercise**			11.281	0.001
Yes	204 (6.5)	2,946 (93.5)		
No	88 (9.8)	814 (90.2)		

**Figure 1:**
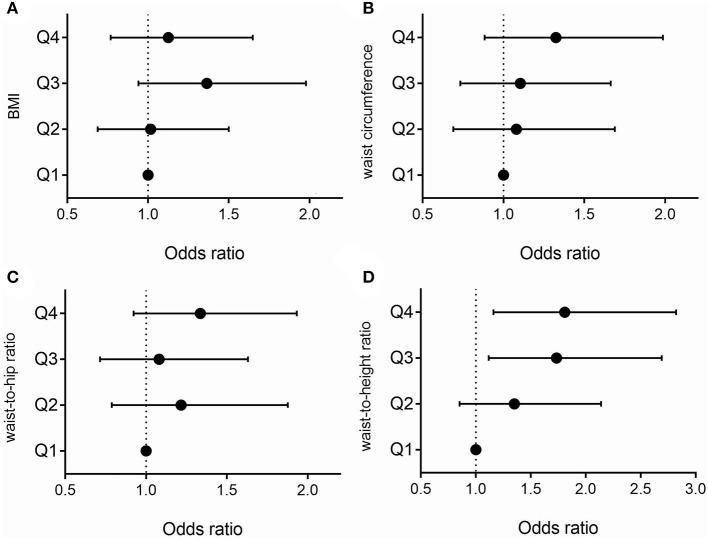
Association between stroke and BMI quartiles **(A)**. Association between stroke and waist circumference quartiles **(B)**. Association between stroke and waist-to-hip ratio quartiles **(C)**. Association between stroke and waist-to-height ratio quartiles **(D)**. Analyses were adjusted for age, sex, area, education, smoking, drinking, hypertension, diabetes, hyperlipidemia, coronary heart disease, family history of stroke, and regular exercise.

Table 2 described the odds ratio and its 95% confidence intervals for the stroke prevalence rate, according to quartiles of BMI, waist circumference, waist-to-hip ratio, and waist-to-height ratio. For BMI (adjusted OR: 1.13, 95% CI: 0.77–1.65) (Figure 1A), waist circumference (adjusted OR: 1.32, 95% CI: 0.88–1.99) (Figure 1B), and waist-to-hip ratio (adjusted OR: 1.34, 95% CI: 0.92–1.93) (Figure 1C), the patients in the highest quartile did not have a higher risk of stroke than those in the lowest quartile. For waist-to-height ratio, the patients in the highest quartile were more likely to have a stroke than those in the lowest quartile (adjusted OR: 1.81, 95% CI: 1.16–2.82) (Figure 1D). The linear trend test showed that the risk of stroke increases with waist-to-height ratio only after adjustment for age, sex, area, education, smoking, drinking, hypertension, diabetes, hyperlipidemia, coronary heart disease, family history of stroke and regular exercise (*p* = 0.006). Similarly, the risk of stroke increased 1.19 (95% CI: 1.04–1.37) times per standard deviation (SD) only when waist-to-height ratio was considered.

**Table 2:** Odds ratio (95% confidence intervals) for stroke prevalence rate, according to the quartiles of BMI, waist circumference, waist-to-hip ratio, and waist-to-height ratio.

**Body fat measuring indexes**	**Quartiles of body fat measuring indexes**				***P* for trend**	**Per SD increase**
	**Q1**	**Q2**	**Q3**	**Q4**		
**BMI (kg/m**^**2**^**)**	<22.19	22.19–24.22	24.23–26.63	≥26.64	—	—
No of events (%)	1,006 (24.8)	1,019 (25.1)	1,011 (25.0)	1,016 (25.1)	—	—
Model 1^*^	1	1.06 (0.73, 1.53)	1.47 (1.04, 2.08)	1.47 (1.04, 2.07)	0.010	1.16 (1.03, 1.30)
Model 2^†^	1	1.14 (0.79, 1.66)	1.59 (1.11, 2.26)	1.57 (1.10, 2.23)	0.005	1.19 (1.06, 1.34)
Model 3^‡^	1	1.02 (0.69, 1.50)	1.36 (0.94, 1.98)	1.13 (0.77, 1.65)	0.380	1.06 (0.93, 1.21)
**Waist circumference (cm)**	<80.3	80.3–84.2	84.3–90.9	≥91.0	—	—
No of events (%)	978 (24.1)	859 (21.2)	1,141 (28.2)	1,074 (26.5)	—	—
Model 1^*^	1	1.42 (0.93, 2.18)	1.98 (1.35, 2.91)	2.80 (1.92, 4.04)	<0.001	1.44 (1.28, 1.63)
Model 2^†^	1	1.23 (0.79, 1.90)	1.40 (0.95, 2.08)	1.91 (1.30, 2.80)	0.123	1.26 (1.11, 1.43)
Model 3^‡^	1	1.08 (0.69, 1.69)	1.10 (0.73, 1.66)	1.32 (0.88, 1.99)	0.721	1.10 (0.96, 1.26)
**Waist-to-hip ratio**	<0.8800	0.8800–0.8946	0.8947–0.9037	≥0.9038	—	—
No of events (%)	1,009 (24.8)	1,000 (24.7)	963 (23.8)	1,080 (26.7)	—	—
Model 1^*^	1	1.07 (0.72, 1.59)	1.52 (1.05, 2.20)	2.24 (1.59, 3.15)	<0.001	1.33 (1.17, 1.51)
Model 2^†^	1	1.17 (0.78, 1.76)	1.23 (0.84, 1.79)	1.70 (1.20, 2.41)	0.005	1.21 (1.07, 1.37)
Model 3^‡^	1	1.22 (0.79, 1.88)	1.08 (0.72, 1.63)	1.34 (0.92, 1.93)	0.152	1.11 (0.98, 1.27)
**Waist-to-height ratio**	<0.4880	0.4880–0.5272	0.5273–0.5651	≥0.5652	—	—
No of events (%)	1,011 (25.0)	1,010 (24.9)	1,017 (25.1)	1,014 (25.0)	—	—
Model 1^*^	1	1.93 (1.25, 3.00)	2.93 (1.94, 4.44)	3.76 (2.51, 5.63)	<0.001	1.52 (1.36, 1.71)
Model 2^†^	1	1.63 (1.05, 2.55)	2.24 (1.47, 3.42)	2.62 (1.73, 3.98)	<0.001	1.36 (1.20, 1.55)
Model 3^‡^	1	1.35 (0.85, 2.14)	1.73 (1.12,2.69)	1.81 (1.16,2.82)	0.006	1.19 (1.04,1.37)

* *Unadjusted*.

† *Adjusted for age and sex*.

‡ *Adjusted for age, sex, area, education, smoking, drinking, hypertension, diabetes, hyperlipidemia, coronary heart disease, family history of stroke, and regular exercise*.

**Figure 2:**
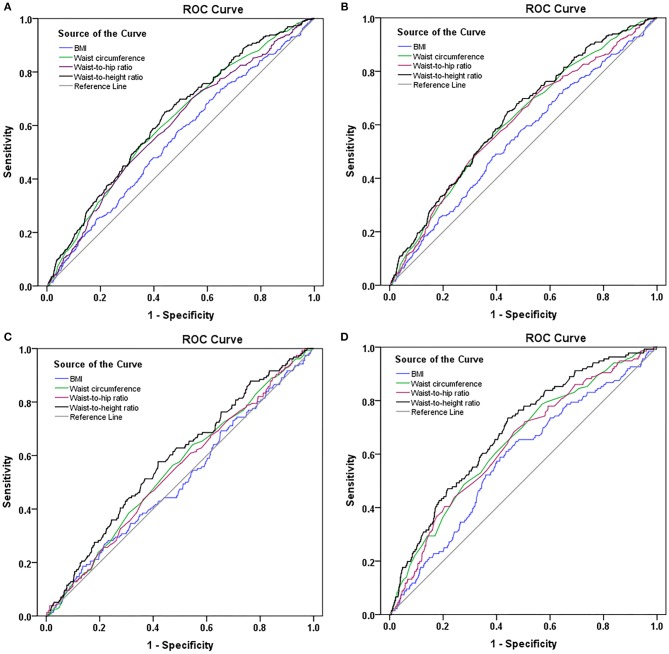
ROC curves of the body fat measuring indexes for stroke **(A)**. ROC curves of the body fat measuring indexes for ischemic stroke **(B)**. ROC curves of the body fat measuring indexes for stroke in males **(C)**. ROC curves of the body fat measuring indexes for stroke in females **(D)**.

Table 3 described the area under the curve and the optimum cut-off point of the body fat measuring indexes. The area under the curve of waist-to-height ratio, waist-to-hip ratio, waist circumference, and BMI for stroke were 0.627 (95% CI: 0.595–0.659), 0.596 (95% CI: 0.593–0.629), 0.612 (95% CI: 0.579–0.644), and 0.548 (95% CI: 0.514–0.583), respectively (Table 3 and Figure 2). The results of ischemic stroke were similar to those of stroke (Supplementary Tables I, II). When the optimum cut-off point of waist-to-height ratio was 0.5331, the corresponding sensitivity was 65.10% and the specificity was 56.00%. The area under the curve of waist-to-height ratio for stroke in females was higher than that in males (0.683 vs. 0.583) (Supplementary Table III). The results of the pairwise comparison of the ROC curves were showed in Supplementary Tables IV–VII.

**Table 3:** The area under the curve and the optimum cut-off point of the body fat measuring indexes for stroke.

**Test variables**	**Area under the curve**	**95% Confidence Interval**		**Optimum cut-off point**	**Sensitivity (%)**	**Specificity (%)**	**Positive predictive value (%)**	**Negative predictive value (%)**
		**Lower Bound**	**Upper Bound**					
BMI	0.548	0.514	0.583	24.25	57.90	51.10	8.42	93.99
Waist circumference	0.612	0.579	0.644	89.50	52.70	64.70	10.38	94.63
Waist-to-hip ratio	0.596	0.593	0.629	0.8919	69.90	45.90	9.12	95.15
Waist-to-height ratio	0.627	0.595	0.659	0.5331	65.10	56.00	10.29	95.38

## Discussion

Since the incidence and prevalence of stroke increased significantly after 39 years of age, our study included only participants over 39 years of age (16). In this study, waist-to-height ratio seemed superior to the other indexes when it was used to indicate the association with the prevalence of stroke or ischemic stroke. The trend test indicated that the higher the waist-to-height ratio, the greater the prevalence of stroke. This conclusion was not applicable to BMI, waist circumference, and waist-to-hip ratio. After adjusting for multiple covariates, only waist-to-height ratio had a significant association with the prevalence of stroke for each 1-SD increase. The possible explanation was that waist-to-height ratio could better describe the distribution of body fat associated with the prevalence of stroke. The degree of body fat accumulation was usually described by body mass index (BMI), but it discriminated poorly between fat and lean mass and did not consider the distribution of body fat (17). Waist-to-hip ratio reflected the proportion of fat around the torso, but increasing evidence indicated that health risk was determined by the relative distribution of fat in the central or abdominal region (18), so excessive waist circumference (WC) appeared to be more of a risk factor for diseases than BMI and waist-to-hip ratio (19, 20). However, the value of the waist circumference was closely related to the height of individuals. The association between WC and diseases might be erroneously estimated without the consideration of height (21). This might explain why waist-to-height ratio was superior to other indexes when it was used to indicate the association with the prevalence of stroke or ischemic stroke.

A meta-analysis indicated that both waist circumference, waist-to-hip ratio, and waist-to-height ratio were positively associated with the risk of stroke (22). Three of the included studies in the meta-analysis were conducted in China. Some studies showed similar results to ours but the others showed different results. This may be due to the different research design of these studies. However, participants recruited from different regions may also play an important role. Jilin Province is a region with a high incidence of stroke. Our study revealed a phenomenon that the relationship between stroke and obesity indicators may be different in areas with high incidence of stroke and areas with low incidence of stroke, and only waist-to-height was positively correlated with stroke in areas with high incidence of stroke. It provided clues for further prospective studies on the relationship between stroke and obesity indicators in areas with high incidence of stroke.

In the current study, the areas under the curve for BMI, waist circumference, waist-to-hip ratio, and waist-to-height ratio were 0.548, 0.612, 0.596, and 0.627, respectively. Xu et al. obtained similar result. They explored the predictive values of BMI, waist circumference, and waist-to-height ratio for ischemic stroke in a population-based, prospective cohort study. The areas under the curve for each index were as follows: 0.586 for waist-to-height ratio, 0.543 for waist circumference, and 0.566 for BMI (23). Although the area under the curve for waist-to-height ratio was not higher than waist circumference after the pairwise comparison of the ROC curves, waist-to-height ratio was more closely related to the prevalence of stroke than waist circumference as waist-to-height ratio demonstrated a higher OR for indicating the association with the prevalence of stroke or ischemic stroke after adjusting for covariates.

Waist-to-height ratio was also of important clinical significance for other diseases. Meseri et al. conducted a cross sectional study to determine the best anthropometric measurement among waist-to-height ratio, BMI, waist-to-hip ratio, and waist circumference associated with high coronary heart disease risk in adults (24). They concluded that waist-to-height ratio was a successful measurement for determining cardiovascular risks. A systematic literature review indicated that waist-to-height ratio was of great value in predicting many non-communicable diseases in the elderly (25).

In the present study, the role of waist-to-height ratio in describing the association with the prevalence of stroke was more significant in females. This sex difference has not been previously reported. A systematic review indicated that the most commonly recommended and used cutoff value of waist-to-height ratio was > 0.5 for both sexes to predict the risk of cardiovascular disease (26). This conclusion might not apply to cerebrovascular disease. Males and females should have different waist-to-height ratio criteria for describing the association with the prevalence of stroke.

High BMI was considered an important risk factor for stroke in clinical practice in China. However, study showed that approximately 36% of the general population had abnormal waist circumference (WC ≥ 80 cm for female and WC ≥ 85 cm for male), but their BMI was normal (18.5 ≤ BMI < 24) (27). Focusing solely on the relationship between BMI and stroke would ignore the risk of stroke in these individuals. BMI, waist circumference, waist-to-hip ratio, and waist-to-height ratio demonstrated different association with the prevalence of stroke. Comparing the four anthropometric indexes and identifying the index that was most closely related to stroke were very important for the primary prevention of stroke in Jilin province. A similar study explored the association between body fat distribution and the risk of incident ischemic stroke (28). Their results showed that waist circumference or waist-to-height ratio was superior to BMI in predicting the risk of ischemic stroke.

Some potential limitations exist in this study. First, the participants were recruited from Jilin province where the prevalence and incidence of stroke were higher than other parts of China, and the conclusion could not be extended to other areas. In addition, our cross-sectional study cannot elucidate the causal relationship between anthropometric indexes and stroke, but can only provide clues to risk factors for further research. Third, lack of consideration of dietary and psychological factors in multivariable logistic analysis is a disadvantage of this study. Finally, self-reported data and the nature of cross-sectional data may have led to recall and reporting biases, which may have affected the accuracy of the results. The strength of this study lies in a representative sampling survey and a high response rate.

## Conclusions

The waist-to-height ratio was more closely related to the prevalence of stroke than body mass index, waist circumference, and weight-to-hip ratio among participants aged ≥ 40 years.

## Data Availability Statement

The datasets generated for this study are available on request to the corresponding author.

## Ethics Statement

The studies involving human participants were reviewed and approved by the human ethics and research ethics committee of the First Hospital of Jilin University. The patients/participants provided their written informed consent to participate in this study.

## Author Contributions

YY and XS: conception and design. F-LZ and HJ: acquisition of the data. PZ: data analysis and drafting the manuscript. Z-NG and YY: critical revision. All of the authors approved the final version for publication.

### Conflict of Interest

The authors declare that the research was conducted in the absence of any commercial or financial relationships that could be construed as a potential conflict of interest.
