# Supplementary material for: Comparison of the Four Anthropometric Indexes and Their Association With Stroke: A Population-Based Cross-Sectional Study in Jilin Province, China
Source: Front Neurol. 2019 Dec 10;10:1304. doi: 10.3389/fneur.2019.01304 (PMC6914861; doi:10.3389/fneur.2019.01304)
Supplement: Supplementary file 1 [file Data_Sheet_1.PDF]

Supplementary Table I. The area under the curve of the body fat measuring indexes for ischemic stroke.

Test variables	Area under the curve	95% Confidence Interval	
		Lower Bound	Upper Bound
BMI	0.548	0.512	0.584
Waist circumference	0.613	0.579	0.647
Waist-to-hip ratio	0.603	0.568	0.638
Waist-to-height ratio	0.628	0.594	0.661

Supplementary Table II. Odds ratio (95% confidence intervals) for ischemic stroke prevalence rate, according to quartiles of BMI, waist circumference, waist-to-hip ratio, and waist-to-height ratio.

Body fat measuring indexes	Quartiles of body fat measuring indexes				P for trend	Per SD increase
	Q1	Q2	Q3	Q4		
**BMI(kg/m²)**	<22.19	22.19-24.21	24.22-26.63	⩾26.64	—	—
No of events	1003(24.9)	986(24.5)	1027(25.5)	1009(25.1)	—	—
Model 1*	1	0.980(0.667,1.439)	1.371(0.960,1.956)	1.417(0.993,2.021)	0.019	1.157(1.024,1.307)
Model 2†	1	1.064(0.718,1.577)	1.483(1.028,2.138)	1.525(1.058,2.198)	0.009	1.194(1.053,1.354)
Model 3‡	1	0.944(0.628,1.420)	1.271(0.865,1.867)	1.095(0.739,1.623)	0.448	1.066(0.932,1.220)
**Waist circumference(cm)**	<80.0	80.0-84.9	85.00-91.9	⩾92.00	—	—
No of events	974(24.2)	1082(26.9)	905(22.5)	1064(26.4)	—	—
Model 1*	1	1.476(0.965,2.258)	2.083(1.375,3.157)	2.823(1.912.4.167)	<0.001	1.450(1.281,1.641)
Model 2†	1	1.224(0.794,1.888)	1.476(0.964,2.260)	1.951(1.306,2.915)	<0.001	1.274(1.116,1.455)
Model 3‡	1	1.059(0.678,1.655)	1.154(0.740,1.799)	1.354(0.886,2.070)	0.102	1.108(0.961,1.278)

**Waist-to-hip ratio**	<0.8800	0.8800-0.8946	0.8947-0.9037	⩾0.9038	—	—
No of events	1004(24.9)	992(24.6)	957(23.9)	1072(26.6)	—	—
Model 1*	1	1.013(0.666,1.539)	1.543(1.047,2.272)	2.309(1.616,3.300)	<0.001	1.347(1.179,1.538)
Model 2†	1	1.103(0.718,1.693)	1.256(0.846,1.866)	1.763(1.222,2.542)	0.003	1.229(1.076,1.405)
Model 3‡	1	1.156(0.730,1.831)	1.108(0.720,1.704)	1.394(0.949,2.049)	0.099	1.128(0.984,1.294)
**Waist-to-height ratio**	<0.4878	0.4878-0.5272	0.5273-0.5648	⩾0.5649	—	—
No of events	998(24.8)	1014(25.2)	1005(25.0)	1008(25.0)	—	—
Model 1*	1	1.987(1.250,3.159)	3.034(1.958,4.711)	3.858(2.514,5.920)	<0.001	1.532(1.356,1.731)
Model 2†	1	1.695(1.058,2.716)	2.330(1.489,3.644)	2.709(1.740,4.217)	<0.001	1.373(1.203,1.567)
Model 3‡	1	1.396(0.859,2.270)	1.793(1.126,2.857)	1.863(1.165,2.980)	0.007	1.198(1.039,1.382)

* Unadjusted.

† Adjusted for age and sex.

‡ Adjusted for age, sex, area, education, smoking, drinking, hypertension, diabetes, hyperlipidemia, coronary heart disease, family history of stroke, and regular exercise.

Supplementary Table III. The area under the curve of the body fat measuring indexes for stroke in different sexes.

Test variables	Area under the curve	95% Confidence Interval	
		Lower Bound	Upper Bound
**Male**			
BMI	0.508	0.459	0.556
Waist circumference	0.546	0.500	0.593
Waist-to-hip ratio	0.540	0.493	0.586
Waist-to-height ratio	0.583	0.537	0.629
**Female**			
BMI	0.578	0.530	0.627
Waist circumference	0.643	0.596	0.690
Waist-to-hip ratio	0.634	0.587	0.681
Waist-to-height ratio	0.683	0.639	0.727

Supplementary Table IV. Pairwise comparison of ROC curves of body fat measuring indexes for stroke. (Z, P)

Indicators	BMI	Waist circumference	Waist-to-hip ratio	Waist-to-height ratio
**BMI**	--	5.202,<0.0001	2.875,0.0040	5.992,<0.0001
**Waist circumference**	5.202,<0.0001	--	1.407,0.1593	1.851,0.0641
**Waist-to-hip ratio**	2.875,0.0040	1.407,0.1593	--	2.484,0.0130
**Waist-to-height ratio**	5.992,<0.0001	1.851,0.0641	2.484,0.0130	--

Supplementary Table V. Pairwise comparison of ROC curves of body fat measuring indexes for ischemic stroke. (Z, P)

Indicators	BMI	Waist circumference	Waist-to-hip ratio	Waist-to-height ratio
**BMI**	--	5.110,<0.0001	3.154,0.0016	5.784,<0.0001
**Waist circumference**	5.110,<0.0001	--	0.867,0.3859	1.710,0.0872
**Waist-to-hip ratio**	3.154,0.0016	0.867,0.3859	--	1.904,0.0569
**Waist-to-height ratio**	5.784,<0.0001	1.710,0.0872	1.904,0.0569	--

Supplementary Table VI. Pairwise comparison of ROC curves of body fat measuring indexes for stroke in males. (Z, P)

Indicators	BMI	Waist circumference	Waist-to-hip ratio	Waist-to-height ratio
**BMI**	--	2.321,0.0203	1.349,0.1772	4.135,<0.0001
**Waist circumference**	2.321,0.0203	--	0.451,0.6523	3.807,<0.0001
**Waist-to-hip ratio**	1.349,0.1772	0.451,0.6523	--	2.589,0.0096
**Waist-to-height ratio**	4.135,<0.0001	3.807,<0.0001	2.589,0.0096	--

Supplementary Table VII. Pairwise comparison of ROC curves of body fat measuring indexes for stroke in females. (Z, P)

Indicators	BMI	Waist circumference	Waist-to-hip ratio	Waist-to-height ratio
**BMI**	--	3.687,0.0002	2.412,0.0158	5.839,<0.0001
**Waist circumference**	3.687,0.0002	--	0.579,0.5626	4.884,<0.0001
**Waist-to-hip ratio**	2.412,0.0158	0.579,0.5626	--	3.059,0.0022
**Waist-to-height ratio**	5.839,<0.0001	4.884,<0.0001	3.059,0.0022	--
